# Prediction of Cross-resistance and Collateral Sensitivity by Gene Expression profiles and Genomic Mutations

**DOI:** 10.1038/s41598-017-14335-7

**Published:** 2017-10-25

**Authors:** Takaaki Horinouchi, Shingo Suzuki, Hazuki Kotani, Kumi Tanabe, Natsue Sakata, Hiroshi Shimizu, Chikara Furusawa

**Affiliations:** 1 Quantitative Biology Center, RIKEN, 6-2-3 Furuedai, Suita, Osaka, 565-0874 Japan; 2 Department of Bioinformatic Engineering, Graduate School of Information Science and Technology, Osaka University, 2-1 Yamadaoka, Suita, Osaka, 565-0871 Japan; 3 Universal Biology Institute, The University of Tokyo, 7-3-1 Hongo, Bunkyo-ku, Tokyo, 113-0033 Japan

## Abstract

In adaptive evolution, an increase in fitness to an environment is frequently accompanied by changes in fitness to other environmental conditions, called cross-resistance and sensitivity. Although the networks between fitness changes affect the course of evolution substantially, the mechanisms underlying such fitness changes are yet to be fully elucidated. Herein, we performed high-throughput laboratory evolution of *Escherichia coli* under various stress conditions using an automated culture system, and quantified how the acquisition of resistance to one stressor alters the resistance to other stressors. We demonstrated that resistance changes could be quantitatively predicted based on changes in the transcriptome of the resistant strains. We also identified several genes and gene functions, for which mutations were commonly fixed in the strains resistant to the same stress, which could partially explain the observed cross-resistance and collateral sensitivity. The integration of transcriptome and genome data enabled us to clarify the bacterial stress resistance mechanisms.

## Introduction

Laboratory evolution of microorganisms is a powerful approach to the elucidation of the nature of evolutionary dynamics^1,2^. Recent advances in measurement technology, including high-throughput sequencing, have enabled us to quantify phenotypic and genotypic changes during laboratory evolution, which have provided valuable information on the mechanisms and principles of adaptive evolution^3–5^. The impact of laboratory evolution has extended beyond the field of evolutionary biology into engineering and medicine. For example, by using laboratory evolution approaches, some candidate mutations that contribute to antibiotic resistance have been identified, and this sheds light on how to control the emergence of antibiotic-resistant strains^6–9^. Laboratory evolution has also become a widely used tool for bioengineering applications^10–13^— to generate cells with improved growth, production titer, and stress tolerance, which are essential for improving industrial microbial production.

The evolutionary adaptation to a specific environment is frequently accompanied by changes in fitness in response to other environments. For example, it was demonstrated that the acquisition of resistance to one antibiotic can give rise to resistance to other drugs simultaneously, which is called cross-resistance, while it can also increase sensitivity to other drugs, which is called collateral sensitivity^14–19^. Such links between changes in fitness affect the course of evolution considerably. This can be utilized for predicting and controlling evolutionary dynamics, such as the suppression of resistance acquisition by use of multiple antibiotics in combination, with collateral sensitivity interactions. Such a “design” of evolutionary dynamics based on the links between fitness changes can contribute to the suppression of emerging multidrug-resistant pathogens^20–22^, and to the development of useful microorganisms for bioproduction based on evolutionary engineering^23^. Although there are several large-scale analyses of phenotype-genotype mapping using microbial laboratory evolution^5,24–26^, these studies were based on evolutionary dynamics under a limited range of environmental conditions. The phenotype-genotype mappings underlying the links between cross-resistance and collateral sensitivity remain largely unknown.

Given the importance of the links between changes in fitness to various environments with an understanding of evolutionary dynamics and medical or engineering applications, we performed high-throughput laboratory evolution of *E*. *coli* cells under 11 different conditions of environmental stress. We used a variety of stressors with different mechanisms of stress, including acids, alcohols, detergents, and so on. Some metabolites useful in bioengineering, which can cause environmental stress in their production processes, were also included. After constructing strains that were resistant to each stressor, we quantified the changes in resistances to other stressors for analyzing the links in the acquisition of fitness. Furthermore, we analyzed the changes in the transcriptomes and genome sequences of these resistant strains to reveal the mechanisms of stress resistance and their links. We analyzed the correlation between these changes in expression and mutations that were commonly fixed in the strains, which were resistant to the same stress. This analysis revealed the mechanisms for some of the stress resistance phenomena, which were validated by introducing the relevant mutations into the genome of the parental strain. The integration of the multilevel phenotypic and genotypic data enabled us to understand a wide range of molecular mechanisms of stress resistance in *E*. *coli* and their interactions.

## Results

### Laboratory evolution under 11 stress conditions and analysis of cross-resistance

**Figure 1:**
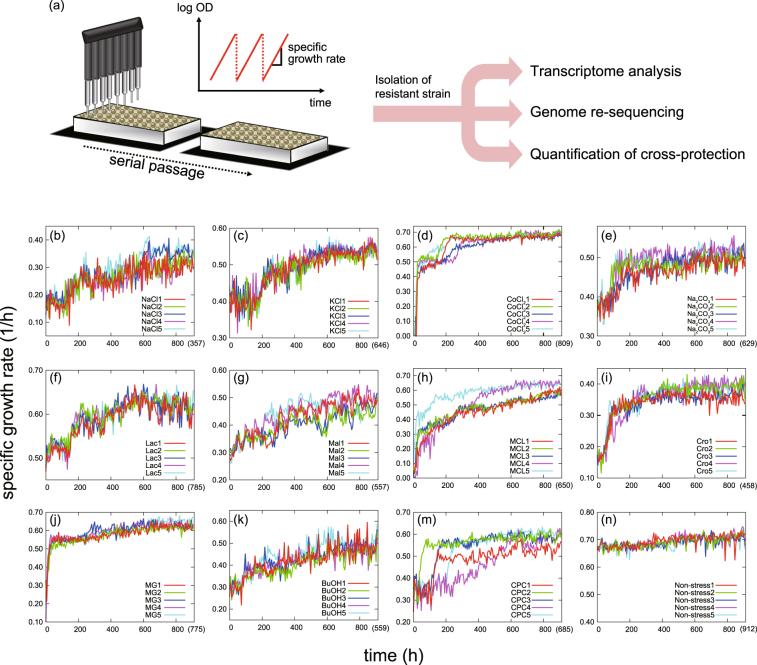
Laboratory evolution under environmental stress. (**a**) Overview of the experimental setting. *E*. *coli* cells were cultured under 11 stress conditions by using the automated culture system. Selected clones from adapted populations were sequenced, and transcriptional profiles were quantified to analyze phenotype-genotype relationships. (**b–n**) Time courses of the specific growth rate in experimental evolution. Five parallel series of experiments were performed. The values in parentheses show the average number of generations at the end point of the culture.

We selected 11 stress conditions that simulated a wide range of environmental stress conditions (Table 1). *E*. *coli* MDS42 cells^27^ were cultured in 200 μL of M9 synthetic medium^28^ with a constant concentration of stressors. The concentrations of these stressors were set to levels that initially decreased the specific growth rate by approximately one-half of the non-stress growth condition. Every 6 hours, a fraction of the cells was transferred to fresh medium containing the stressor. The transfer volume was adjusted to maintain the final cell concentration below a threshold, and to maintain the cells in exponential phase. The specific growth rate was determined using the initial and final cell concentrations, which were used as the measures of fitness under these stress conditions (Fig. 1a). To evaluate the reproducibility of the evolutionary pathways for each stress, 5 independent culture lines were propagated in parallel. For this high-throughput laboratory evolution, we used a custom-developed automated system^29^, by which we could maintain numerous independent culture series in a fully automated manner. After 906 hours of propagation, we observed considerable increase in the specific growth rates under all 11 stress conditions (Fig. 1b to m). In addition to the cultures with stressors, we maintained the cells in synthetic medium without adding stressors (Fig. 1n). In these cultures without stressors, slight increases in the specific growth rate were also observed, although these increases were substantially smaller than those observed under the stress conditions. At each end point, we isolated a single clone from the culture, and confirmed that the growth rate of the isolated clone was identical to that of the corresponding population in the endpoint culture. Throughout the study, phenotypic and genotypic changes were analyzed using the isolated resistant strains.

**Table 1:** List of stress conditions used for the laboratory evolution.

Stressor	Abbreviation	Concentration	Description of stress
Sodium chloride	NaCl	400 mM	Inorganic salt
Potassium chloride	KCl	210 mM	Inorganic salt
Cobalt chloride	CoCl_2_	16 μM	Heavy metal
Sodium carbonate	Na_2_CO_3_	32.5 mM	Alkali
L-Lactate	Lac	40 mM	Organic acid
L-Malate	Mal	30 mM	Organic acid
Methacrylate	MCL	8.75 mM	Unsaturated carboxylic acid
Crotonate	Cro	50 mM	Unsaturated carboxylic acid
Methylglyoxal	MG	350 μM	Ketoaldehyde
*n*-butanol	BuOH	1.25%	Alcohol
Cetylpyridinium chloride	CPC	4.8 μM	Surfactant

**Figure 2:**
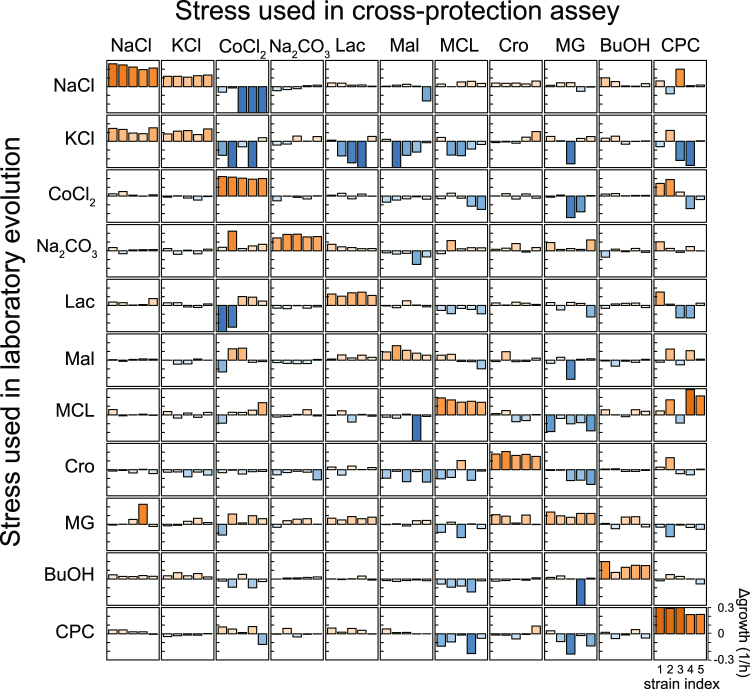
Quantification of cross-resistance and collateral sensitivity. The vertical axis of each figure shows the difference in the specific growth rate compared with the parental strains, while the horizontal axis corresponds to the index of five resistant strains constructed independently. Each column of figures corresponds to the data obtained under the same stress condition, while each row indicates the stress used for the laboratory evolution. The color of the bars represents the sign of the growth difference and its magnitude.

To explore evolutionary cross-resistance and collateral sensitivity for each resistant strain, we measured the specific growth rate under all the stress conditions. In Fig. 2, the growth rates of the resistant strains relative to the parental strain are provided (all the growth rates are provided in Supplementary Table S1). The results demonstrated that the stress-resistant strains often showed cross-resistance and collateral sensitivity to multiple stress conditions. A clear phenomenon of converse cross-resistance was observed among strains resistant to NaCl and KCl stress, i.e., NaCl-resistant strains exhibited considerable KCl resistance and vice versa. This fact suggested that at least some of the mechanisms of resistance were common to the NaCl- and KCl-resistant strains. Also, *n*-butanol (BuOH)-resistant strains showed small increases in the growth rate under the conditions of NaCl and KCl stress. This weak cross-resistance may also indicate common mechanisms of resistance, as will be discussed subsequently. For other cases, cross-resistance was occasionally asymmetric, which suggested that the mechanisms of cross-resistance and sensitivity were independent, or that there are hierarchical relationships between the mechanisms of resistance or sensitivity. For example, methylglyoxal (MG)-resistant strains exhibited resistance to lactate (Lac), while Lac-resistant strains did not affect MG-resistance substantially. This asymmetric cross-resistance could be explained by a hierarchical structure of the mechanisms of resistance. In the pathways of the metabolic degradation of MG, one potential intermediate is Lac^30^, and thus, the simultaneous development of Lac resistance in the MG-resistant strains might be required. In contrast, MG is not involved in the pathway of Lac degradation, and thus, Lac resistance would not necessarily be accompanied by MG resistance.

### Changes in expression profiles in the stress-resistant strains

**Figure 3:**
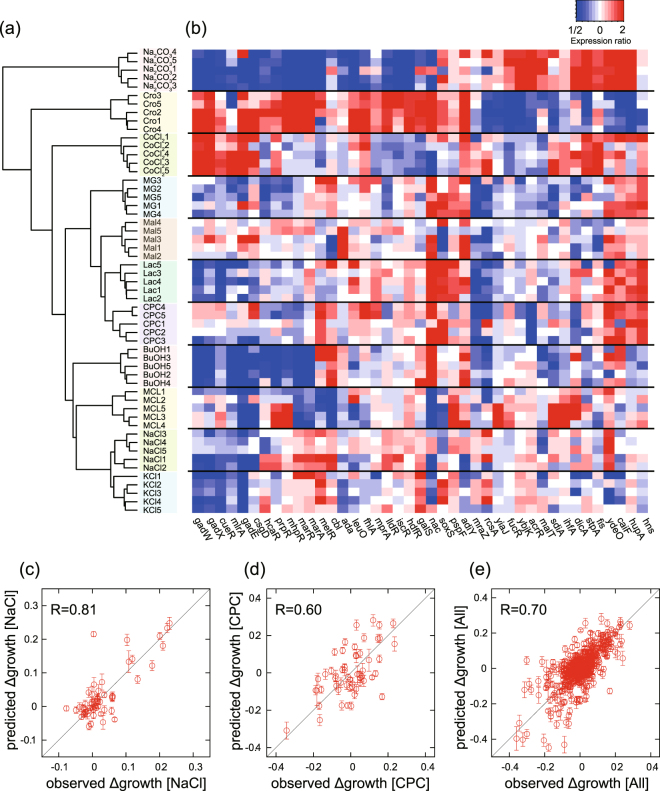
Transcriptomic changes in the resistant strains. (**a**) Hierarchical clustering of overall changes in expression in the resistant strains. The changes in expression were calculated by dividing the expression level of the genes in the resistant strains by that of the parental strains, wherein the expression was quantified under the corresponding stress condition. (**b**) Changes in the expression of the transcriptional factors. Representative transcriptional factors having larger variance in expression changes across the resistant strains were plotted. The changes in expression more than 2-fold or less than 1/2 are shown in the same color with 2 and 1/2, respectively. (**c**–**e**) Prediction of the growth rate using transcriptomic changes. Comparisons between observed and predicted growth rates under (**c**) NaCl stress, (**d**) CPC stress; (**e**) all data were calculated by fitting using the following 15 genes: *tbpA*, *appB*, *ydiH*, *gadE*, *sbp*, *aldB*, *asr, marC*, *proW*, *tktB*, *nac*, *thiC*, *ydhZ*, *acs*, and *gcd*. Only test data, which were not used for fitting, are plotted. The error bars in the y-axis were obtained using predicted growth rate that was calculated from 10,000 different sets of test data and training data. For the details on the prediction of growth rate, see Methods.

To elucidate the mechanisms of resistance to the various stress conditions and their links, we performed transcriptome analysis of all the resistant strains that we had constructed, by using microarray experiments (all transcriptome data are provided in Supplementary Table S2). The results demonstrated that the changes in expression, which occurred during the long-term culture, were the most similar among the strains that developed in the same stress environment, as shown in the data of the hierarchical clustering analysis of the transcriptomes (Fig. 3a). To characterize the similarity in the changes in the transcriptome of these resistant strains, the changes in expression of representative transcriptional factors (TFs) are illustrated in Fig. 3b. As shown, the stress-resistant strains exhibited similar expression profiles of the TFs within each clade. For example, the strains resistant to NaCl and KCl showed highly similar changes in expression. This similarity in the expression profiles of the TFs of the strains resistant to NaCl and KCl could be the reason for the similarity in the changes in stress resistance (Fig. 2).

To further analyze the correlation between changes in the transcriptome and stress resistance, we used a simple, previously published^9^ mathematical model for predicting the resistance using the obtained gene expression profiles. Briefly, we assumed that the changes in the specific growth rates under the stress conditions are represented by a linear combination of log-transformed changes in expression during the long-term culture. Then, we sought to determine the optimal number and combination of genes with the highest prediction accuracy by using cross-validation and a genetic algorithm (see Methods for details). As a result, we found that the combination of 15 to 20 genes offered the highest prediction accuracy on average (Supplementary Fig. S1a). Figure 3c–e show examples of the prediction accuracy by the linear model with 15 genes (see legend of Fig. 3 for details; all fitting results are shown in Supplementary Fig. S1). In this analysis, the coefficients of the linear model were estimated by fitting the training data for each stress environment, while the plotted data are the test data that were not used for fitting. The estimated growth rate under the stress conditions corresponded to the observed values, indicating that this linear model could predict the change in stress-resistance phenotype by a relatively small number of genes. This analysis enabled us to isolate the genes, whose expression changes provided the most relevant information for predicting stress resistance (Supplementary Fig. S1b). For example, *tbpA*, which encodes the thiamine ABC transporter, was selected as one of the most informative genes for representing the observed changes in resistance. *tbpA* was specifically upregulated in Na_2_CO_3_-resistant strains, while it was downregulated in the strains resistant to NaCl, KCl, and Cro (Supplementary Fig. S2a). Similarly, *ydiH*, which encodes a predicted protein with unknown function, was commonly upregulated in the strains resistant to CoCl_2_ and Cro, while it was downregulated in several resistant strains (Supplementary Fig. S2b). In this study, we successfully screened genes, whose expression changes were highly correlated with the acquisition of resistance, which could contribute to highly accurate descriptions of complex evolutionary dynamics.

It should be noted here that, the prediction of resistance based on gene expression can also represent cross-resistance and collateral sensitivity. For example, the strains resistant to NaCl and KCl exhibited collateral sensitivity to CoCl_2_ (see Fig. 2). In the prediction based on gene expression, as shown in Fig. 3, this decrease in CoCl_2_ resistance was represented by the downregulation of *tbpA*. For another example, the cross-resistance to BuOH and CPC observed in MCL-resistant strains was explained by the downregulation of *aldB*, which encodes aldehyde dehydrogenase. Although these results were obtained based on the correlation between the changes in stress resistance and the levels of gene expression, which do not necessarily correspond to a causal relationship, they might help in proposing hypotheses for experimental verification.

### Mutations fixed in the stress-resistant strains

**Figure 4:**
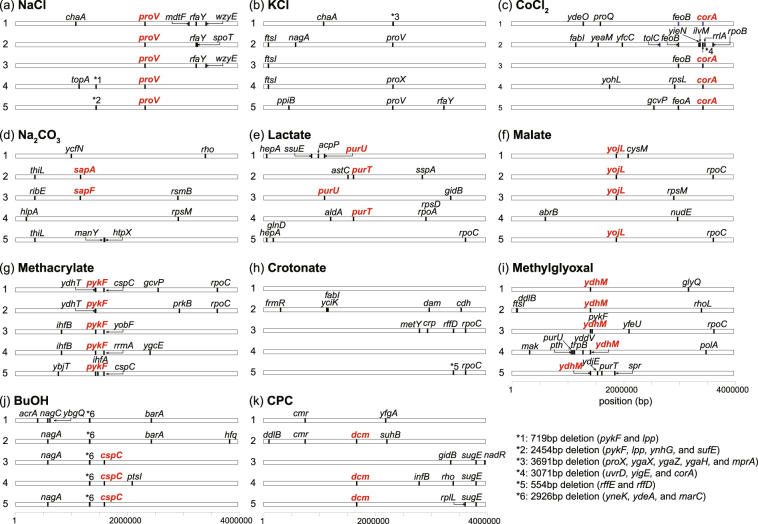
Map of the mutations that were identified in the resistant strains. Coordinates are relative to the reference MDS42 genome. The representative mutations that were commonly identified in the strains resistant to the same stress are highlighted by letters in red font, and whose effects on the corresponding stress resistance were evaluated, as shown in Fig. 5. The detailed information on the identified mutations is provided in Table S3.

The mutations that were identified in the resistant strains are shown in Fig. 4, and the detailed information about the mutations is provided in Supplementary Table S3. Less than 10 mutations were fixed in each of the resistant strains. We also sequenced two strains that were maintained without exposure to the stressors (Fig. 1n), and found that a relatively small number of mutations were fixed in these control strains.

**Figure 5:**
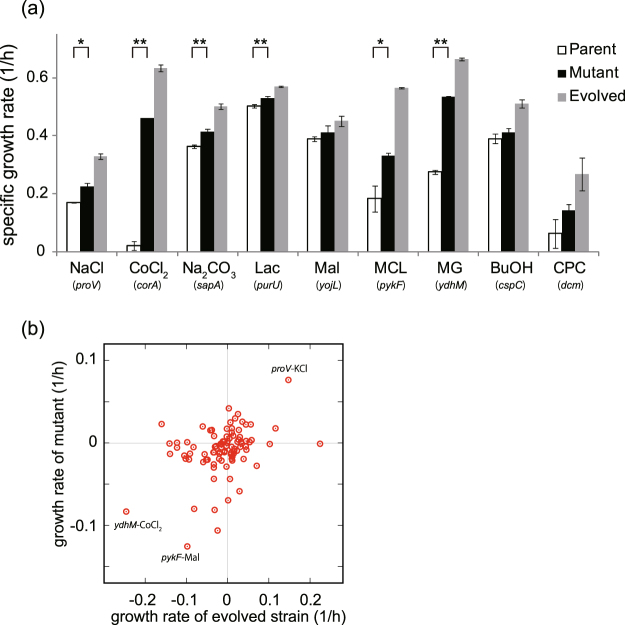
Growth rates of the site-directed mutants. One of the mutations, which were commonly identified in the strains resistant to a given stress condition (highlighted by letters in red font in Fig. 4) were introduced back into the parental strain. (**a**) Growth rate of the mutants in the corresponding environment used in the laboratory evolution. For each mutant, the names of the mutant genes are shown. Error bars indicate standard deviations that were calculated from three independent cultures. Statistical analysis was performed using t-test. Significance was accepted at the *P < 0.05 and **P < 0.01 levels. (**b**) Relation between the changes in growth rate of the resistant strains and the mutants under various environmental conditions. The horizontal axis shows the observed cross-resistance or collateral sensitivity in the resistant strains (shown in Fig. 2), while the vertical axis represents that observed in the corresponding mutants. The growth rates of the mutants in the environment in which the corresponding resistant strains were constructed have been excluded from this figure, and only the data of cross-resistance or collateral sensitivity are shown. Some labels of data points have been overlaid to show the relationship between the mutations and the environments. For example, the point with “*pykF*-Mal” represents the changes in the growth rate of the *pykF* mutant and the MLC-resistant strain #3, in which the *pykF* mutation was identified under Mal stress.

We identified several genes and gene functions, for which mutations were commonly fixed in the resistant strains, suggesting that these mutations contributed to resistance. To verify the possible contribution of these mutations to stress resistance, we introduced some of the common, identified mutations (highlighted by letters in red font in Fig. 4) into the genome of the parental strain, and quantified the change in growth rate under corresponding stress conditions (Fig. 5a). The introduced mutations are highlighted in Supplementary Table S3. We discuss some examples of the relation between the acquisition resistance and the changes in phenotype or genotype in the subsequent paragraphs. A full description of the discussions is presented in Supplementary Text S1.

The first example of the common mutations is the mutation in the *proU* ABC transporter, which was identified in all the NaCl-resistant strains and 4 KCl-resistant strains (Fig. 4a and b). All of these mutations were frameshift mutations, suggesting that they disrupted the activity of the *proU* transport system. The *proU* operon (*proVWX*) encodes a binding protein-dependent transport system, which is essential for the uptake of osmoprotectants, such as glycine betaine, and is known to be upregulated in response to osmotic stress^31^. Our expression analysis showed that the expression of the *proU* operon was upregulated substantially (>50 times) in response to the initial addition of NaCl as a stressor; however, after long-term culture, the expression levels decreased when the cells acquired NaCl resistance (Supplementary Fig. S3). These results suggested that the programmed upregulation of the *proU* operon in response to osmotic stress is not beneficial in an environment without osmoprotectants such as those that we used. Instead, the upregulation of the *proU* operon suppresses cell growth presumably because of energy consumption by the transporter activity. Thus, the disruption of the activity of the *proU* transporter could contribute to active cellular growth under NaCl stress. To support this hypothesis, we introduced a frame-shift mutation that was identified in one of the NaCl-resistant strains into the parental strain using site-directed mutagenesis, and confirmed that this mutation increased cell growth under NaCl stress (Fig. 5a).

Another example is that all the strains resistant to MG stress had mutations related to *ydhM* (*nemR*), which encodes the repressor of N-ethylmaleimide reductase, as shown in Fig. 4i (4 strains had mutations in the upstream region, and one strain had a mutation in the coding region of *ydhM*). We confirmed that the introduction of mutations in the upstream region of *ydhM* caused a considerable increase in growth rate under MG stress (Fig. 5a). The level of *ydhM* expression was upregulated substantially (approximately 100 times) by the exposure of the parental strain to MG stress, while similar expression levels were maintained after the acquisition of MG resistance through laboratory evolution (Supplementary Fig. S4a). Interestingly, after the evolutionary adaptation to MG stress, the upregulation of *ydhM* expression was observed even without the exposure to MG stress (Supplementary Fig. S4a), which suggested that the identified mutations in *ydhM* in the resistant strains served to “assimilate” the stress response into a genetically encoded, invariant, stress-resistance phenotype. This genetic assimilation^32,33^ might contribute to the robustness of the stress-resistance phenotype by altering the reaction norm^34^ to MG stress. *ydhM* expression controls the expression of *gloA*, which is involved in the pathway of the degradation of MG to lactate, and is known to contribute to MG resistance^30^. There is a clear correlation between the levels of *ydhM* and *gloA* expression in the resistant strains (Supplementary Fig. S4b), which suggests that the observed MG resistance was caused by an increase in MG degradation through the upregulation of *gloA* expression.

The detailed description of the other common mutations that were identified in the resistant strains is provided in Supplementary Text S1. We demonstrated that the identified common mutations contributed to the acquisition of resistance, as shown in Fig. 5a. It should be noted that for understanding the mechanism whereby such mutations affected the stress-resistance phenotype, in addition to identifying genomic mutations, the information on changes in expression levels during the adaptive evolution was also highly valuable, as observed in the abovementioned cases of *proU* and *ydhM*.

Interestingly, the common mutations that were identified in the resistant strains partially accounted for the observed cross-resistance and collateral sensitivity, as shown in Fig. 2. For each of the mutant strains, we quantified the growth rates under all 11 stress conditions that we used, in order to evaluate whether these mutations could cause the cross-resistance or collateral sensitivity (all data are provided in Supplementary Table S4). Figure 5b shows the relationship between the growth rates of the mutant strains and those of the corresponding resistant strains having mutations that were introduced into the mutant strains. The data points of the growth rates measured under the 11 stress conditions were overlaid. The weak correlation (R = 0.36; p < 10^−3^), as shown in Fig. 5b, indicated that cross-resistance and collateral sensitivity could be partly explained by the mutations that we examined. For example, the mutation in *proV* in the NaCl-resistant strains was suggested to be responsible for the observed cross-resistance to KCl stress, which was consistent with the abovementioned discussion on the contribution of the mutation in *proV* to NaCl or KCl stress. Another example is the collateral sensitivity to Mal stress observed in MLC-resistant strains. In the MCL-resistant strains, mutations were commonly fixed in *pykF* (Fig. 4), which encodes a pyruvate kinase that catalyzes the conversion of phosphoenolpyruvate (PEP) to pyruvate in the central metabolic pathway. The disruption of *pykF* activity caused an accumulation of PEP, which could result in increasing metabolic flux of oxaloacetate and Mal^35^. This metabolic redirection could make MLC-resistant strains sensitive to Mal stress. The mutation in *ydhM* that was commonly identified in the MG-resistant strains caused a decrease in CoCl_2_ resistance, which could correspond to the fact that one of the MG-resistant strains (strain #1) exhibited a decrease in CoCl_2_ resistance, although the underlying molecular mechanism remains unclear.

## Discussion

The laboratory evolution experiments combined with organism-wide analyses of phenotype and genotype enabled us to understand the mechanisms of adaptive evolution. In this study, we demonstrated that by using the high-throughput system that we developed for laboratory evolution, we could construct strains of *E*. *coli* resistant to various stress conditions by long-term culture. Using this system, we could culture up to 44 microplates (96 or 384 wells) simultaneously, and thus, more than 10,000 independent culture series could be maintained in a fully automated manner. This system allowed us to trace evolutionary dynamics under various environmental conditions and initial conditions (for example, all *E*. *coli* strains in the single-gene knockout library^36^) with a large number of replicate experiments.

In this system for laboratory evolution, we cultured the cells on a relatively small scale (for example, 200 μL) using microplates. One might question whether such a small-scale culture results in a small population size, which causes the fixation of random drifts and the accumulation of neutral mutations. Our data suggested that this was not the case. We confirmed that the population size was maintained at more than 10^5^ cells. The ratio of synonymous to non-synonymous substitutions in all the resistant strains was relatively small in comparison with that of neutral mutations, suggesting that the fixation of a majority of substitutions was driven by evolutionary selection pressure. The fact that, for many stress conditions, the strains resistant to the same stress possessed mutations in common also supports the idea of evolutionary selection.

The genome-wide analyses of expression and resequencing showed common phenotypic and genotypic changes in the strains resistant to the same stress. Notably, our results demonstrated that the combined use of the analyses of transcriptome and genome resequencing greatly accelerated our interpretation of how *E*. *coli* strains acquired stress resistance, and how cross-resistance and collateral sensitivity emerged. For example, mutations related to the *proU* operon found in all the NaCl-resistant strains were thought to neutralize the deleterious regulatory program. The use of only sets of fixed mutations in resistant strains often makes it difficult to clarify the mechanisms for the stress resistances.

Adaptive evolution to environmental changes is a phenomenon that involves changes in the genome, transcriptome, metabolome, and so on, which implies that a complex interaction network is involved. One possible strategy for understanding such complex dynamics is to analyze large-scale data for each hierarchical layer, and then, to integrate the analyses to extract the essential components for the phenotypic and genotypic changes. The present study is one of the first to conduct a genome-wide analysis of the strains constructed by high-throughput laboratory evolution, and we succeeded in extracting the phenotypic or genotypic changes responsible for stress resistance and their interactions. We believe that such large-scale data of laboratory evolution will help us to reveal the principles of evolutionary dynamics, and provide valuable information on the rational design of industrially useful microbial strains.

## Methods

### Laboratory evolution

The *Escherichia coli* strain that is free of IS elements, MDS42^27^, was purchased from Scarab Genomics, and used as the parental strain for the laboratory evolution. The use of the strain free of IS elements could ensure the reliability of the resequencing analysis, since the determination of the precise position of IS elements using high-throughput sequencing is often difficult. For serial transfer culture, 200 μL of modified M9 medium^28^ was used with 5 g/L glucose as the carbon source. The cells were cultured in 96-well microplates (Corning Inc.) with agitation at 300 rotations/min at 34 °C. All cultures were performed using the automated culture system^29^ (Fig. 1a) consisting of a Biomek® NX span-8 laboratory automation workstation (Beckman Coulter, Tokyo, JP) in a clean booth connected to a microplate reader (FilterMax F5; Molecular devices), a shaker incubator (STX44; Liconic), and a microplate hotel (LPX220, Liconic). The movie of this automated culture system for laboratory evolution is available on YouTube (https://www.youtube.com/watch?v = 4k6qCN7ppsk). The cells were diluted into fresh medium every 6 hours. The cells were maintained in the exponential phase by adjusting the initial cell concentration in each dilution so as to attain a final cell concentration of less than 10^7^ cells per well, as determined by measuring the optical density at 620 nm (OD_620_). Before laboratory evolution under stress conditions, the cells were cultured without exposure to the stressors for 96 hours (approximately 90 generations) to acclimatize them to the M9 medium. The specific growth rate was calculated based on the initial and final cell concentrations in each dilution. After the evolution experiments, the cells obtained were single-cloned cells; they were stored as glycerol stocks at −80 °C, and used for further analysis. The quantification of the specific growth rates of the constructed or genetically manipulated strains (Fig. 2 and Fig. 5) was performed after 60 hours of preculture (approximately 30 to 60 generations) while maintaining the cells in the exponential phase. For the preculture, the same culture conditions as those of the laboratory evolution experiments with the corresponding stressor were used.

### Transcriptome analysis using microarray technology

The cells were precultured for 60 hours under the same culture conditions as those of the laboratory evolution experiments with the corresponding stressor. Then, 5 × 10^7^ cells in the exponential phase were killed immediately by adding an equal volume of ice-cold ethanol containing 10% (w/v) phenol. After that, the cells were harvested by centrifugation, and stored at −80 °C before RNA extraction. Total RNA was isolated and purified from the cells using an RNeasy mini kit with on-column DNase digestion (Qiagen, Hilden, Germany) in accordance with the manufacturer’s instructions. The quality of the purified RNA was evaluated using the Agilent 2100 Bioanalyzer with an RNA 6000 Nano kit (Agilent Technologies). The purified RNA was stored at −80 °C prior to transcriptome analysis. Microarray experiments were performed using the custom-designed Agilent 8 × 60 K array for *E*. *coli* W3110, in which 12 probes were prepared for each gene. One hundred nanograms of purified total RNA was labeled with Cyanine3 (Cy3) using the Low Input Quick Amp WT Labeling Kit (One-color; Agilent Technologies) in accordance with the manufacturer’s instructions. After confirming the yields (>825 ng) and specific activities (>15 pmol/μg) of the Cy3-labeled cRNAs using NanoDrop ND-2000, the labeled cRNAs (600 ng) were fragmented, and then, hybridized to the microarray for 17 h with rotation at 10 rpm at 65 °C in a hybridization oven (Agilent Technologies). Washing and scanning of the microarrays were performed in accordance with the manufacturer’s instructions. Microarray image analysis was performed using Feature extraction version 10.7.3.1 (Agilent Technologies). The background-corrected intensity values were normalized using the quantile normalization method^37^. In order to use only quantitatively reliable data, genes with low expression levels (less than 100 a.u. for all strains) were excluded from the subsequent analysis (approximately 60% of genes were retained). After the exclusion of the genes with low expression, more than 99% of the expression ratios between the biological triplicate data were confirmed to be within the range of 1/1.3 to 1.3.

### Predicting stress resistance based on levels of gene expression

To examine the contribution of the changes in gene expression in response to the stress resistance, a simple mathematical model was constructed to predict resistance using the obtained gene expression profiles^9^. Here, it was assumed that the stress resistance, which was quantified by the change in growth rate after the addition of the stressors, was determined as a function of the levels of gene expression, and any direct effect of the mutations on the resistance was neglected. Furthermore, for simplicity’s sake, nonlinear effects and cross terms of the changes in gene expression were neglected. Thus, the following simple linear model was assumed for predicting the change in growth rate based on the expression levels of *N* genes:

1 $${\rm{\Delta }}{g}_{j}^{k}=\sum _{{\rm{i}}=1}^{{\rm{N}}}{\alpha }_{i}^{k}{X}_{ij}+{\beta }^{k}$$

${\rm{\Delta }}{g}_{j}^{k}$ indicates the changes in growth rate in the *j* ^th^ strain for the *k-* ^th^ stress, ${X}_{ij}$ is the log_10_-transformed expression level of the *i-* ^th^ gene in the *j-* ^th^ strain after standardization to zero mean and unit variance, and ${\alpha }_{i}^{k}$ and ${\beta }^{k}\,$are fitting parameters. The number of genes analyzed in this study was much larger than the number of the pieces of growth rate data, and the use of all expression data for fitting resulted in overfitting giving rise to a meaningless prediction of the growth rate. To avoid overfitting and to obtain the optimal number of genes with the highest prediction accuracy, the cross-validation method was used, in which the data set were separated into training data, which was used for parameter fitting, and test data, which was used to verify the prediction accuracy. When *N* was large, the prediction accuracy for the test data became small because of overfitting, while the accuracy became small when *N* was small, since the linear combination of expression was insufficient to represent the changes in the fitness data. In this analysis, a 5-fold cross validation method was used. Specifically, the resistant strains were randomly partitioned into 5 equal-sized subgroups; 1 subgroup was used as the test dataset for validation, and the remaining 4 subgroups were used for fitting.

The expression levels of the genes in the same operon are generally correlated, which can cause problems in the procedures of gene selection. Thus, in each operon, the genes with the highest value of average level of expression in all the samples were selected, and used for fitting. In addition, since the change in the expression of a relatively invariant gene dominated the experimental error, the genes whose variance of the changes in expression among the resistant strains and the parental strain was below a given threshold were excluded. After selection using these criteria, 413 genes remained, for analysis.

*N* genes used for the fitting were selected using a simple genetic algorithm (GA) without crossover, in which the coefficient of correlation between the predicted and observed changes in the growth rate in the training datasets was used as the fitness function. As an initial population, 1,000 sets of *N* genes were randomly selected, and the fitness of the sets was calculated. Then, gene sets within the top 5% of genes with the highest fitness were selected as the parent sets for the next generation, from which mutant sets were generated by randomly replacing a single gene. Three hundred cycles for the mutant sets were iterated, and the gene sets with the highest fitness were selected to obtain sets of a small number of genes, whose expression levels could represent changes in the resistance and susceptibility to drugs. The selection of gene sets were repeated using 10,000 different training datasets prepared by randomly partitioning the total dataset to obtain the frequency of the genes selected using the GA, as shown in Supplementary Fig. S1b. The fitting was performed by using a custom-designed C program.

### Preparation of genomic DNA

The precultures were prepared by culturing stock strains in 200 μL of modified M9 medium without stressors in 96-well microplates for 23 h with agitation at 34 °C. The precultured cells were diluted to 3 × 10^−5^ as determined by measuring the OD_600_ in 10 mL of fresh modified M9 medium in test tubes. Cell culture was performed at 34 °C for 23 h with agitation at 150 strokes min^−1^ using water-bath shakers (Personal-11, Taitec Co.), and the OD_600_ values were confirmed to reach more than 1.0. Rifampicin (final concentration of 300 μg/mL) was subsequently added, and the culture was continued for a further 3 h to inhibit the initiation of DNA replication. The cells were collected by centrifugation at 20,000 × *g* for 5 min at 25 °C, and the pelleted cells were stored at −80 °C prior to the purification of genomic DNA. Genomic DNA was isolated and purified using a Wizard^®^ Genomic DNA Purification Kit (Promega) in accordance with the manufacturer’s instructions. To increase the purity of genomic DNA, additional phenol extractions were performed before and after the RNase treatment step. The quantity and purity of the genomic DNA were determined by measuring the absorbance at 260 nm, and calculating the ratio of absorbance at 260 and 280 nm (A_260/280_) using NanoDrop ND-2000 spectrophotometer, respectively. As a result, the A_260/280_ values of all the samples were confirmed to be greater than 1.7. The purified genomic DNA was stored at −30 °C prior to use.

### Genome sequence analyses using Illumina HiSeq System

Genome sequence analyses were performed using the Illumina HiSeq System. A 150-bp paired-end library was generated according to the Illumina protocol, and sequenced using Illumina HiSeq. In this study, 58 samples with different barcodes were mixed, and then, sequenced on two lanes, resulting in approximately 300-fold coverage on an average.

The quality of the sequence data was first assessed using FastX-Toolkit 0.0.13.2 (http://hannonlab.cshl.edu/fastx_toolkit), and the raw reads were trimmed using PRINSEQ^38^, whereby both ends with quality scores lower than Q20 were trimmed. The potential nucleotide differences were validated using BRESEQ version 0.28^39^. For structural variations, the sequence reads were analyzed using Quake^40^, and *de novo* assembly was performed using Soapdenovo^41^. The contigs were mapped on the genome using Blast to screen candidates for indels. The *de novo* assembly near the candidate sites were aligned to the genome using T-coffee^42^. Finally, the presence of an indel was confirmed visually.

### Construction of strains with deletion and single-nucleotide substitution mutations

To construct mutant strains, as shown in Fig. 5, identified mutations were introduced into the parental strain using a markerless gene replacement method^43^. Briefly, to construct DNA fragments with deleted coding regions, the upstream flanking regions of the start codon were amplified by PCR using genomic DNA of the parental strain as the template with forward primers containing the *Eco*RI site and reverse primers containing overlapping sequences with the downstream flanking regions of the stop codon. The downstream flanking regions were amplified by PCR with forward primers containing overlapping sequences with the upstream flanking regions and reverse primers containing the *Kpn*I site. After purification using the MinElute PCR Purification Kit (Qiagen), the PCR products were combined by overlap extension PCR. To construct the DNA fragments that introduced an identified mutation, DNA fragments were amplified by PCR using genomic DNA of each resistant strain, wherein the mutation was identified. Each DNA fragment was purified using the MinElute PCR Purification Kit, and then, cloned into the suicide plasmid pST76-K^43^ (The plasmid was kindly provided by Dr. György Pósfai, Biological Research Centre of the Hungarian Academy of Sciences, Hungary). After the verification of the sequences of the DNA fragments by Sanger sequencing, transformation, integration into the genome of the parental strain, replacement stimulated by double-strand breaks, and plasmid curing were performed in accordance with a previously reported method^43^. After the construction of the mutant strains, corresponding genomic regions were amplified by PCR, and then, verified by Sanger sequencing of the PCR products directly.

### Data availability

The normalized data of the microarrays have been deposited in the GEO repository with the accession code GSE89746, and are provided in Table S2, wherein the biological triplicate data for checking the reproducibility of the analysis, i.e., gene expression data obtained from different cultures of the parental strain without the exposure to stress, are also provided. The raw sequence data of genome sequence analyses are available in the DDBJ Sequence Read Archive under the accession number DRA005229.

## Electronic supplementary material

Supplementary Information

Supplementary Table S1

Supplementary Table S2

Supplementary Table S3

Supplementary Table S4
