# Supplementary material for: Prediction of Cross-resistance and Collateral Sensitivity by Gene Expression profiles and Genomic Mutations
Source: Sci Rep. 2017 Oct 25;7:14009. doi: 10.1038/s41598-017-14335-7 (PMC5656584; doi:10.1038/s41598-017-14335-7)
Supplement: Supplementary file 1 — Supplementary Information [file 41598_2017_14335_MOESM1_ESM.pdf]

Supplementary Information:

Prediction of Cross-resistance and Collateral Sensitivity by Gene Expression profiles and Genomic Mutations

Takaaki Horinouchi[1], Shingo Suzuki[1], Hazuki Kotani[1], Kumi Tanabe[1], Natsue Sakata[1], Hiroshi Shimizu[2], Chikara Furusawa[13*]

[1]Quantitative Biology Center, RIKEN, 6-2-3 Furuedai, Suita, Osaka 565-0874, Japan
[2]Department of Bioinformatic Engineering, Graduate School of Information Science and Technology, Osaka University, 2-1 Yamadaoka, Suita, Osaka 565-0871, Japan
[3]Universal Biology Institute, The University of Tokyo, 7-3-1 Hongo, Bunkyo-ku, Tokyo 113-0033, Japan

Supplementary Note: Detailed description of common mutations identified in resistant strains.

The number of mutations identified in the resistant strains is shown in Fig. 4, and detailed information about the mutations is presented in Supplementary Table S3. Below, for each stress we discuss the relationship between resistance acquisition, genome mutations and gene expression changes.

Sodium chloride (NaCl) resistant strains

See Main Text.

Potassium chloride (KCl) resistant strains

In KCl resistant strains, in addition to mutations in *proU* operon as described in Main Text, we found three resistant strains have mutations in the coding region of *ftsI*, which is involved in the cell division process. Since *ftsI* is an essential gene, we did not evaluate the effect of the identified *ftsI* mutations by introducing them into the parent strain. The role of *ftsI* mutations in KCl resistance remains unclear.

Cobalt chloride ($CoCl_2$) resistant strains

All resistant strains to $CoCl_2$ stress had mutations in *corA* [1], which encodes a transporter mediating influx of Mg^{2+}, Ni^{2+}, and Co^{2+}. The identified mutations included frame-shift and deletion of ORF,

suggesting that the disruption of *corA* activity contributed to $CoCl_2$ resistance. This hypothesis was supported by the fact that the introduction of the *corA* mutation found in a $CoCl_2$ resistant strain into the parent strain significantly increased the growth under $CoCl_2$ stress (Fig. 5a). Four out of five $CoCl_2$ resistant strains also had mutations in *feoA*/*feoB* encoding ferrous iron transporters[2]. Even though the role of FeoA/FeoB in cobalt uptake has not been demonstrated[3], these identified *feoA*/*feoB* mutations suggested that FeoA/FeoB transporters are involved in Co^{2+} transport.

Sodium carbonate (Na_2CO_3) resistant strains

Two out of five Na_2CO_3 resistant strains had mutations in *sapABCDF* operon, which encode Sap (Sensitive to antimicrobial peptides) ABC importer[4]. To evaluate the effect of the mutations, we introduced *sapA* mutation in its coding region (found in Na_2CO_3-2 resistant strain) into the parent strain, and confirmed that it significantly increased the growth under Na_2CO_3 stress. Sap transporter is known to contribute alkali stress resistance in *Sinorhizobium meliloti*, root nodule bacteria[5], and thus our result might suggest that Sap transporter is involved in alkali stress resistance also in *E. coli*.

L-Lactate (Lac) resistant strains

Four Lac resistant strains (Lac-1, 2, 3, and 4) had mutations either *purT* or *purU* genes, which are involved in purine biosynthesis[6]. In *Lactobacillus lactis*, it was suggested that perturbing purine biosynthesis is related to multi-stress resistance by changing 3',5'-bispyrophosphate (ppGpp) concentration[7], which is known as a master regulator of the stringent response to amino acid starvation. Thus, the identified mutations in *pur* genes might be related to the control of the stringent response, although the details still remain unclear. We confirmed that the introduction of the mutation in the *purT* coding region significantly increased the growth under Lac stress (Fig. 5a).

L-Malate (Mal) resistant strains

Four Mal resistant strains (Mal-1, 2, 3, and 5) had mutations in the promoter region of *yojL* (*apbE*). The expression levels of *yojL* in these four strains significantly increased (Fig. S5), probably due to the mutations in the promoter region. *yojL* encodes a periplasmic lipoprotein which is involved in thiamine biosynthesis[8], and also it is thought to play a role in assembly or maintenance of iron-sulfur clusters[9]. These mutations commonly fixed in *yojL* promoter region suggests their contribution to Malate resistance by up-regulating its expression level. However, we could not observe a significant growth rate increase under Mal stress when one of these mutations was introduced in the parent strain (Fig. 5a).

Methacrylate (MCL) resistant strains

All resistant strains to MCL had mutations in *pykF*, which encodes a pyruvate kinase that catalyzes

the conversion of phosphoenolpyruvate (PEP) into pyruvate in the central metabolic pathway. All these mutations in *pykF* were nonsynonymous single nucleotide substitutions, which might suggest that the change of enzymatic activity contribute to the methacrylate resistance. To verify this, we introduced the mutation found in a MCL-3 resistant strain into the parent strain, and confirmed that it significantly increased the growth rate under methacrylate stress (Fig. 5a). Although the mechanism how *pykF* mutations contribute to MCL resistance remains unclear, controlling intracellular accumulation of pyruvate caused by MCL stress might be related to the mechanism of resistance. MCL is known to inhibit the enzymatic activity of pyruvate formate-lyase (Pfl) that converts pyruvate into acetyl-CoA and formate[10], and thus MCL stress can cause an increase in intra-cellular pyruvate. Since the PEP/pyruvate concentration ratio is an important parameter to control various metabolic pathways, including glucose uptake via sugar phosphotransferase system (PTS)[11], the decrease of PykF activity by the mutations might play a role in rebalancing the disrupted PEP/pyruvate ratio by MCL stress.

Crotonate (Cro) resistant strains

No common mutation was identified in Cro resistant strains.

Methylglyoxal (MG) resistant strains

See Main Text.

***n*-butanol (BuOH) resistant strains**

Three BuOH resistant strains (BuOH-3, 4, and 5) had mutations in the coding region of *cspC*, and we confirmed that one of these mutations can slightly increase the BuOH stress resistance (Fig. 5a). CspC is a constitutively produced member of the CspA family of RNA-binding proteins, which increases RpoS expression level presumably by stabilizing its mRNA[12]. RpoS is a central regulator of the general stress response whose up-regulation leads to growth arrest[12]. We found that the *rpoS* mRNA expression increased in response to BuOH stress addition, while it relaxed to the original levels in BuOH resistant strains (Fig. S6). This result suggests that the growth rate increase under BuOH stress can be partially explained by the down-regulation of *rpoS* caused by the *cspC* mutations. Furthermore, all BuOH resistant strains had deletion of region including *yneK*, *ydeA*, and *marC* genes. Interestingly, disruption of *marC* was also identified in isobutanol resistant *E. coli* strains obtained by laboratory evolution[13,14]. Although *marC* is a poorly characterized gene whose function is unknown, these results suggest a positive effect of *marC* disruption on butanol resistance.

Cetylpyridinium chloride (CPC) resistant strains

Three out of five CPC resistant strains (CPC-2, 4, and 5) had mutations in *dcm*, which encodes a DNA cytosine methyltransferase. To verify the effect of *dcm* mutations, we introduced the nonsynonymous single nucleotide substitutions found in CPC-5 strain into the parent strain. This mutant strain exhibited a slight increase of growth rate under the CPC stress (Fig. 5a), although the difference was not statistically significant. This result might suggest that the mutation in *dcm* has a weak fitness gain effect in the CPC stress environments. The mechanism for CPC resistance by these *dcm* mutations remains unclear. A recent study demonstrated that the deletion of *dcm* contributes to antibiotic resistance through up-regulation of *sugE* encoding multidrug efflux transporters[15]. In three of five CPC resistant strains (CPC-3, 4, and 5), mutations are found in the promoter region of *sugE*, which suggests that these resistant strains acquired CPC resistance by activating the SugE efflux transporter. The fact that the expression levels of *sugE* significantly increased in CPC-3, 4, and 5 strains and weakly increased in CPC-2 strain supported this hypothesis (Fig. S7).

References

1. Wang, S. Z., Chen, Y., Sun, Z. H., Zhou, Q. & Sui, S. F. Escherichia coli CorA periplasmic domain functions as a homotetramer to bind substrate. *J. Biol. Chem.* **281,** 26813–26820 (2006).
2. Cartron, M. L., Maddocks, S., Gillingham, P., Craven, C. J. & Andrews, S. C. Feo - Transport of ferrous iron into bacteria. in *BioMetals* **19,** 143–157 (2006).
3. Barras, F. & Fontecave, M. Cobalt stress in Escherichia coli and Salmonella enterica: molecular bases for toxicity and resistance. *Metallomics* **3,** 1130 (2011).
4. Harms, C. *et al.* Identification of the ABC protein SapD as the subunit that confers ATP dependence to the K+-uptake systems TrkH and TrKG from Escherichia coli K-12. *Microbiology* **147,** 2991–3003 (2001).
5. Lin, D. X., Tang, H., Wang, E. T. & Chen, W. X. An ABC transporter is required for alkaline stress and potassium transport regulation in Sinorhizobium meliloti: RESEARCH LETTER. *FEMS Microbiol. Lett.* **293,** 35–41 (2009).
6. He, B., Shiau, A., Choi, K. Y., Zalkin, H. & Smith, J. M. Genes of the Escherichia coli pur regulon are negatively controlled by a repressor-operator interaction. *J. Bacteriol.* **172,** 4555–4562 (1990).
7. Rallu, F., Gruss, A., Ehrlich, S. D. & Maguin, E. Acid- and multistress-resistant mutants of Lactococcus lactis: Identification of intracellular stress signals. *Mol. Microbiol.* **35,** 517–528 (2000).
8. Beck, B. J. & Downs, D. M. The apbE gene encodes a lipoprotein involved in thiamine synthesis in Salmonella typhimurium. *J. Bacteriol.* **180,** 885–891 (1998).
9. Skovran, E. & Downs, D. M. Lack of the ApbC or ApbE protein results in a defect in Fe-S cluster

metabolism in Salmonella enterica serovar typhimurium. *J. Bacteriol.* **185,** 98–106 (2003).

10. Plaga, W., Vielhaber, G., Wallach, J. & Knappe, J. Modification of Cys-418 of pyruvate formate-lyase by methacrylic acid, based on its radical mechanism. *FEBS Lett.* **466,** 45–48 (2000).
11. Cunningham, D. S. *et al.* Pyruvate kinase-deficient Escherichia coli exhibits increased plasmid copy number and cyclic AMP levels. *J. Bacteriol.* **191,** 3041–3049 (2009).
12. Battesti, A., Majdalani, N. & Gottesman, S. The RpoS-Mediated General Stress Response in Escherichia coli*. *Annu. Rev. Microbiol.* **65,** 189–213 (2011).
13. Atsumi, S. *et al.* Evolution, genomic analysis, and reconstruction of isobutanol tolerance in Escherichia coli. *Mol. Syst. Biol.* **6,** 449 (2010).
14. Minty, J. J. *et al.* Evolution combined with genomic study elucidates genetic bases of isobutanol tolerance in Escherichia coli. *Microb. Cell Fact.* **10,** 18 (2011).
15. Militello, K. T., Mandarano, A. H., Varechtchouk, O. & Simon, R. D. Cytosine DNA methylation influences drug resistance in Escherichia coli through increased sugE expression. *FEMS Microbiology Letters* **350,** 100–106 (2014).

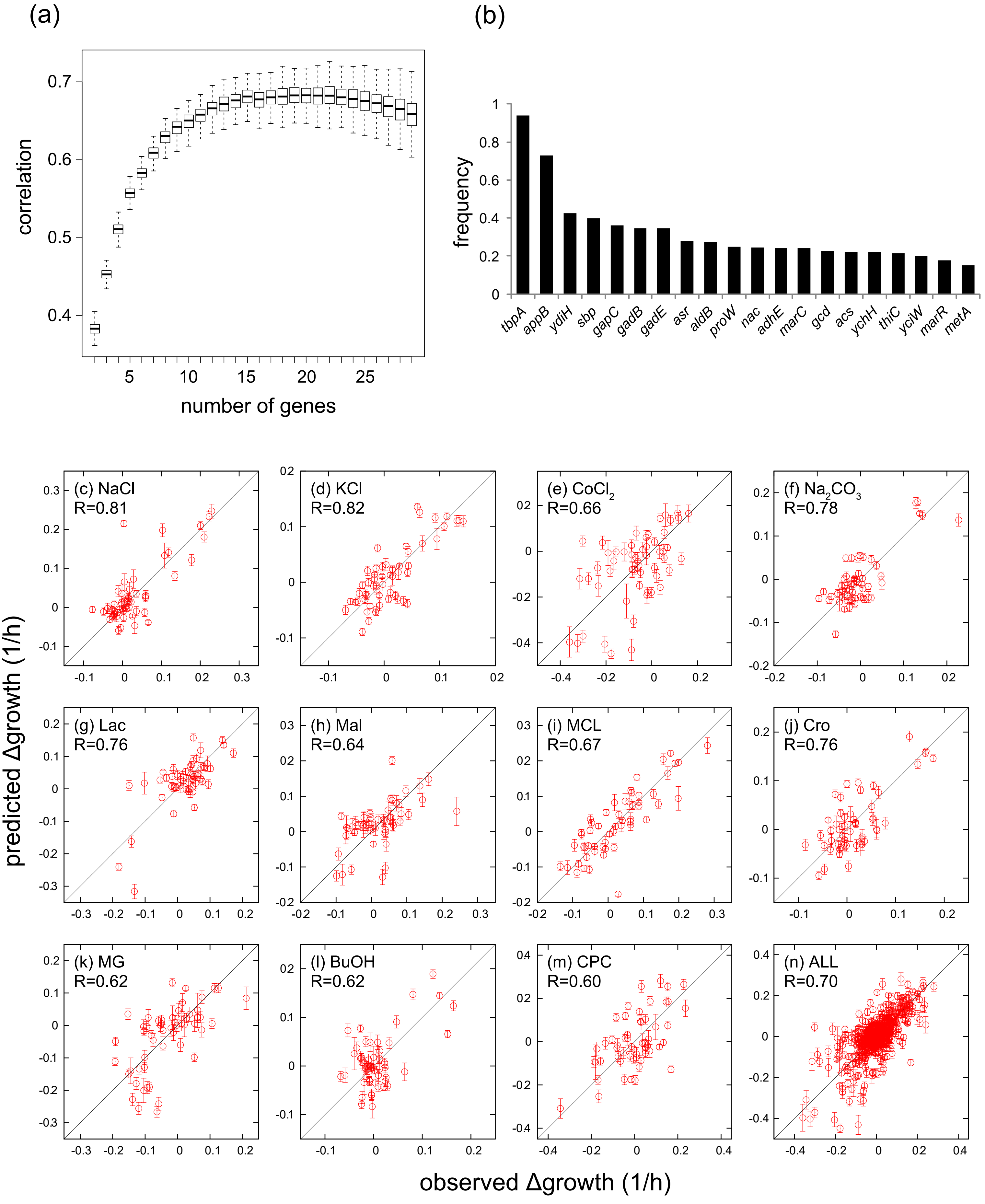

Horinouchi et al, Figure S1

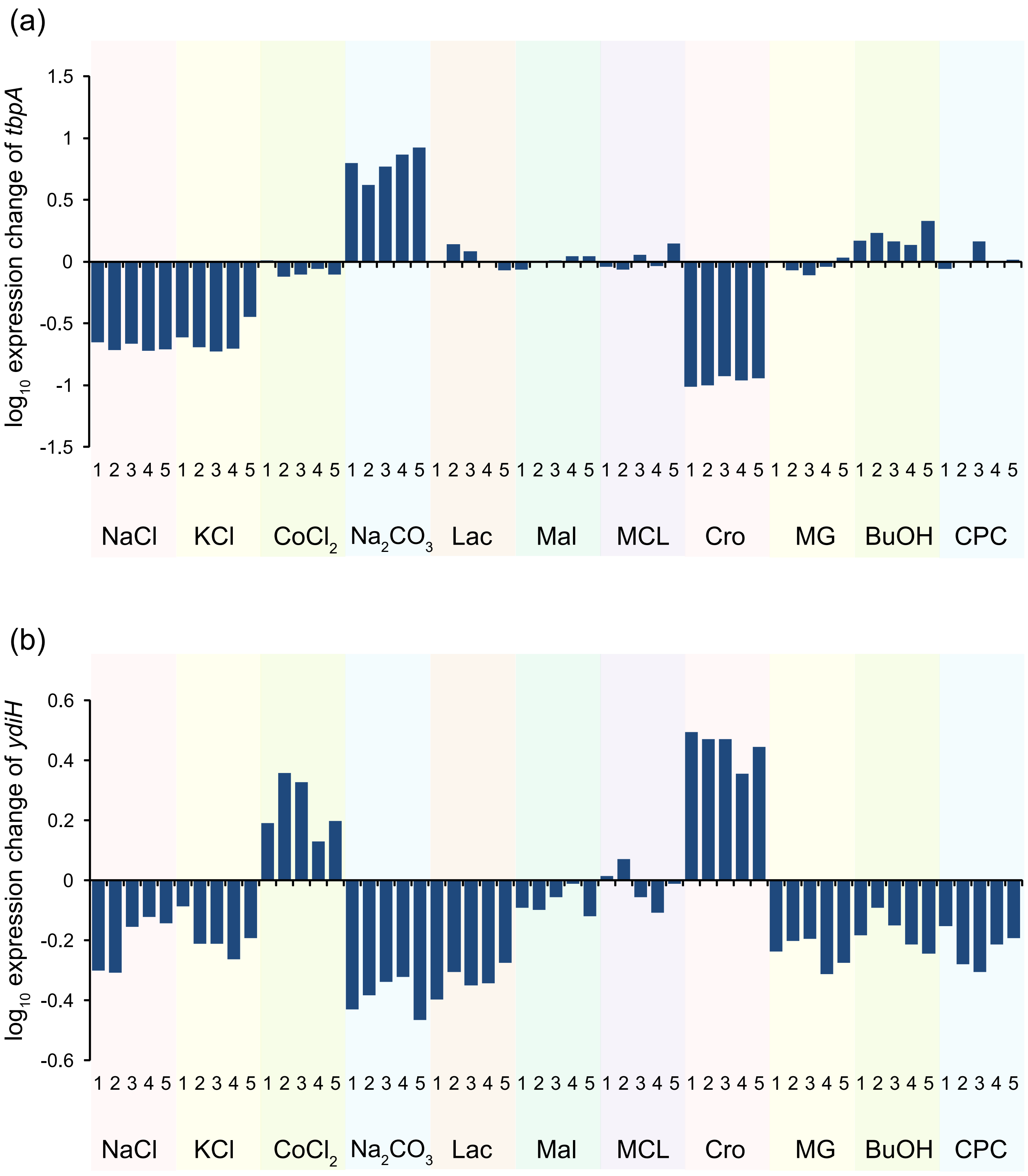

Horinouchi et al, Figure S2

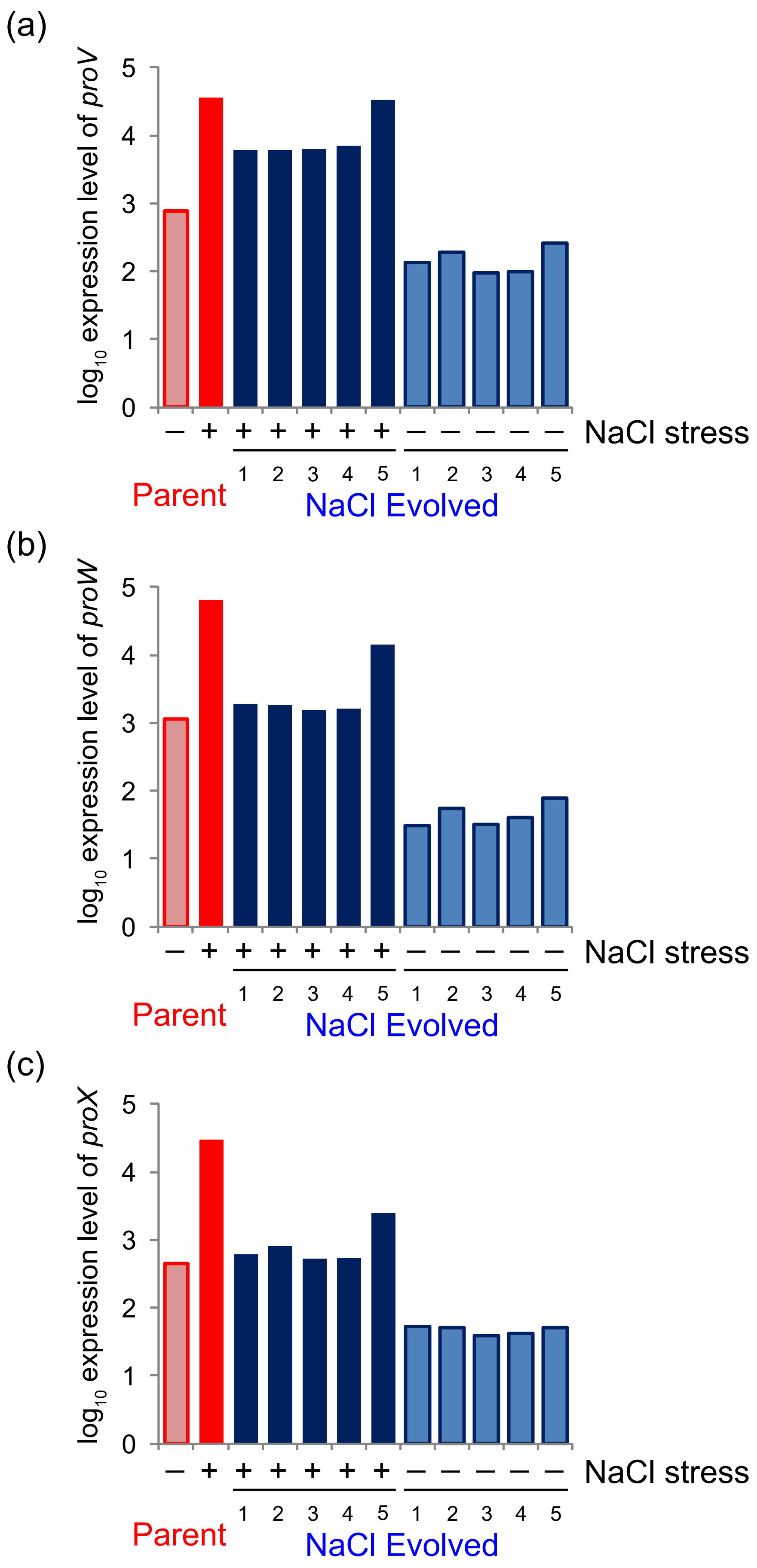

Horinouchi et al, Figure S3

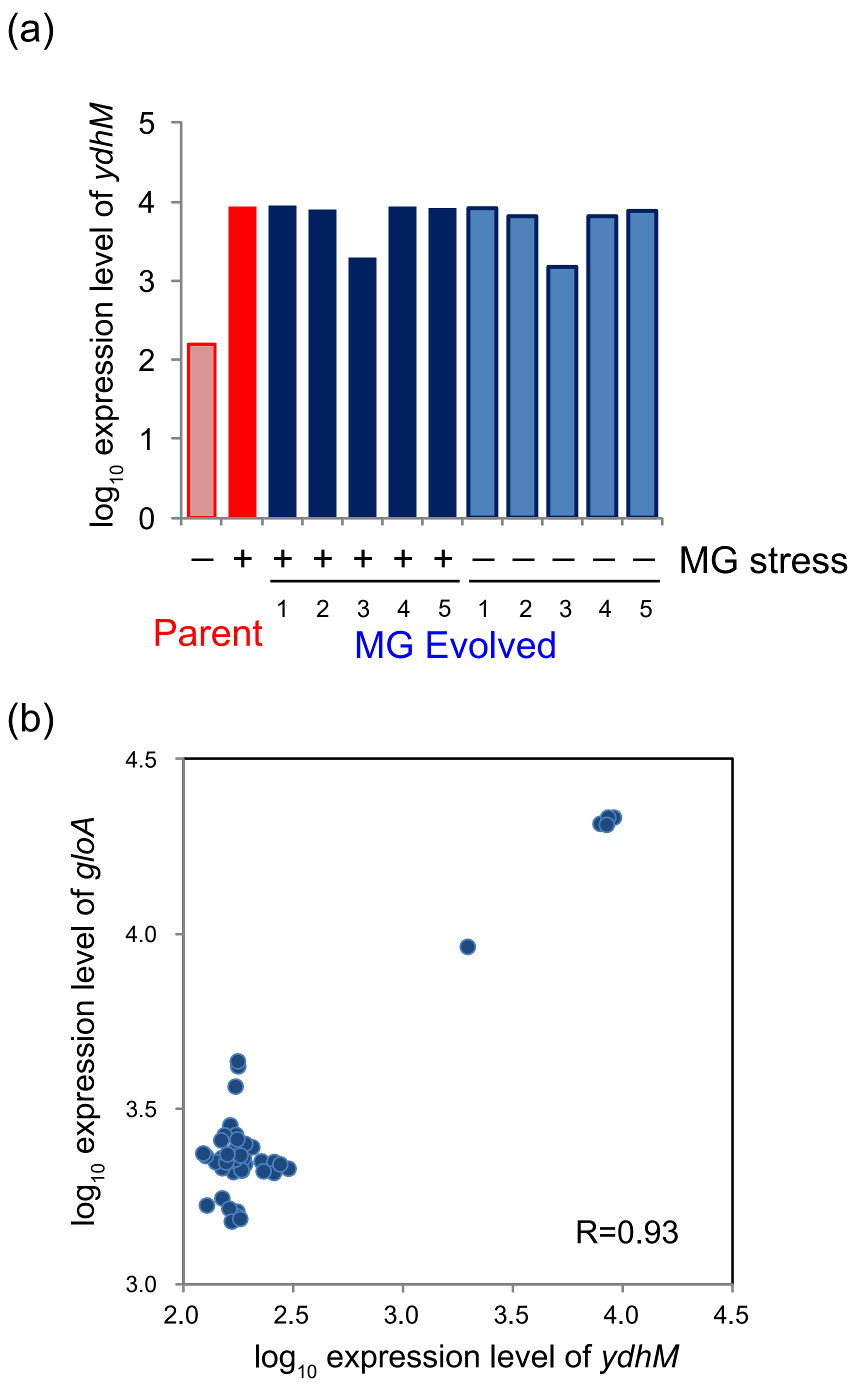

Horinouchi et al, Figure S4

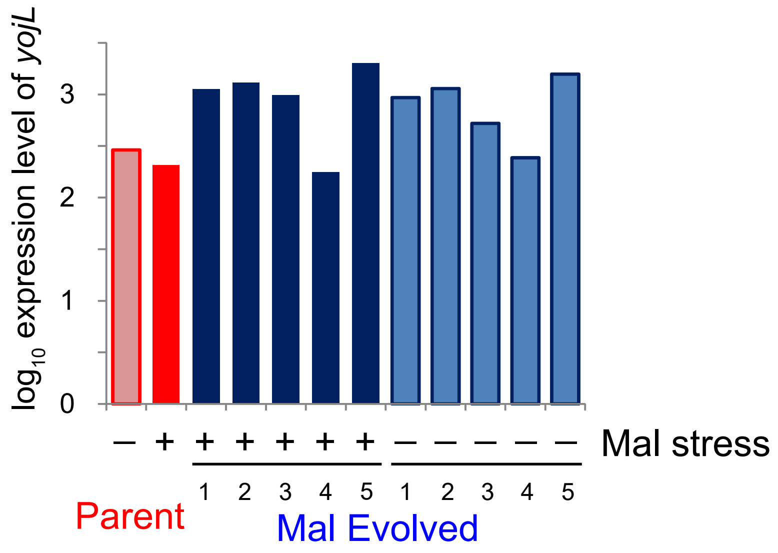

Horinouchi et al, Figure S5

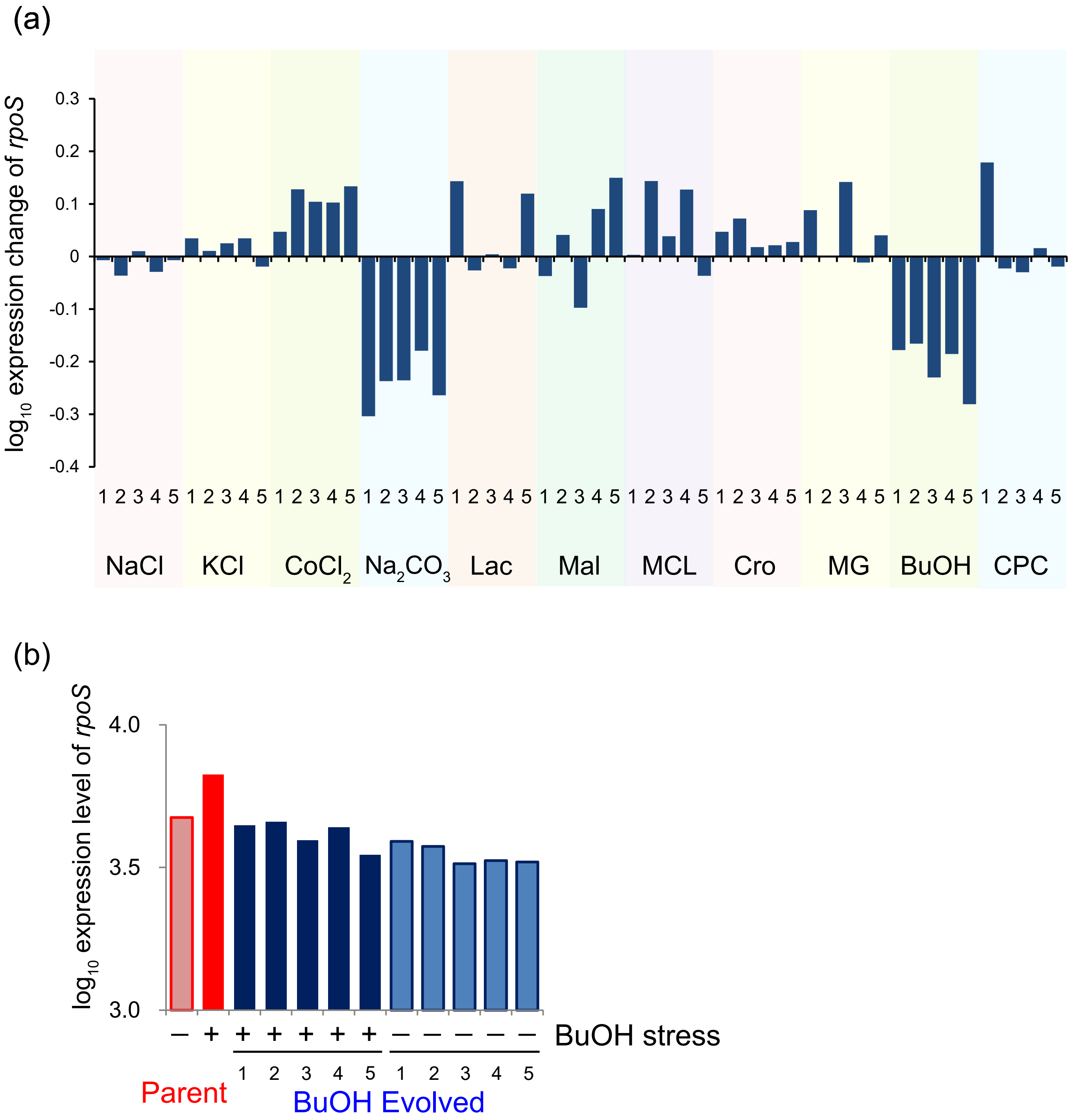

Horinouchi et al, Figure S6

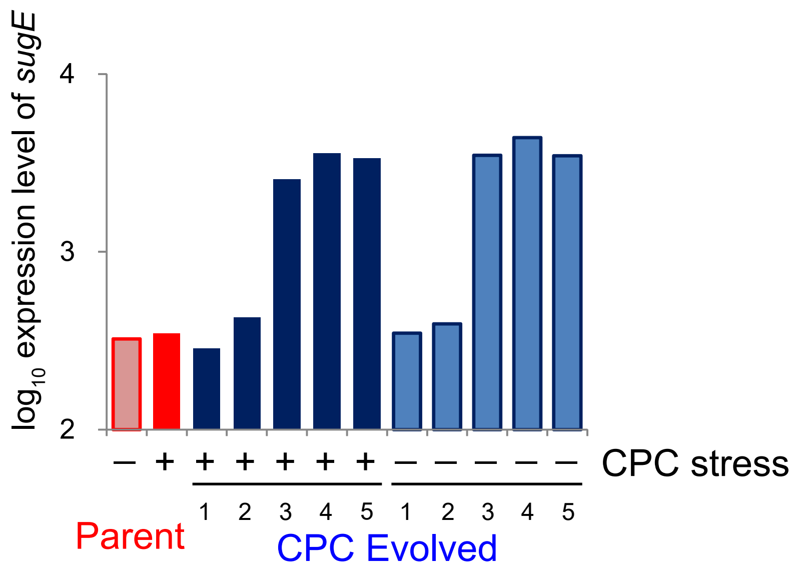

Horinouchi et al, Figure S7
